# Reliability of pleth variability index in predicting preload responsiveness of mechanically ventilated patients under various conditions: a systematic review and meta-analysis

**DOI:** 10.1186/s12871-019-0744-4

**Published:** 2019-05-08

**Authors:** Tianyu Liu, Chao Xu, Min Wang, Zheng Niu, Dunyi Qi

**Affiliations:** 1 Key Laboratory of Anesthesia and Analgesia, Xuzhou Medical University, Xuzhou, Jangsu China; 2 Department of Anesthesiology, Affiliated Hospital of Xuzhou Medical University, Xuzhou, Jangsu China

**Keywords:** Pleth variability index, Preload responsiveness, Mechanically ventilated patients, Meta-analysis

## Abstract

**Background:**

Goal-directed volume expansion is increasingly used for fluid management in mechanically ventilated patients. The Pleth Variability Index (PVI) has been shown to reliably predict preload responsiveness; however, a lot of research on PVI has been published recently, and update of the meta-analysis needs to be completed.

**Methods:**

We searched PUBMED, EMBASE, Cochrane Library, Web of Science (updated to November 7, 2018) and the associated references. Relevant authors and researchers had been contacted for complete data.

**Results:**

Twenty-five studies with 975 mechanically ventilated patients were included in this meta-analysis. The area under the curve (AUC) of receiver operating characteristics (ROC) to predict preload responsiveness was 0.82 (95% confidence interval (CI) 0.79–0.85). The pooled sensitivity was 0.77 (95% CI 0.67–0.85) and the pooled specificity was 0.77 (95% CI 0.71–0.82). The results of subgroup of patients without undergoing surgery (AUC =0.86, Youden index =0.65) and the results of subgroup of patients in ICU (AUC =0.89, Youden index =0.67) were reliable.

**Conclusion:**

The reliability of the PVI is limited, but the PVI can play an important role in bedside monitoring for mechanically ventilated patients who are not undergoing surgery. Patients who are expanded with colloid may be more suitable for PVI.

**Electronic supplementary material:**

The online version of this article (10.1186/s12871-019-0744-4) contains supplementary material, which is available to authorized users.

## Background

Goal-directed fluid therapy has proven benefits for the hemodynamic stability of perioperative and shock patients. Some recent studies have reported that moderate intraoperative volume expansion, and adequate maintenance of cardiac output (CO) can reduce the complications after surgery and the time spent in the intensive care unit (ICU) [1–3]. Inappropriate fluid administration is often harmful to patients; thus, accurate detection of the patient’s hemodynamics can effectively improve the patient’s prognosis (such as decrease in serum lactate, the length of stay in hospital and incidence of postoperative organ complications) [4–6].

A pulse oximeter is a noninvasive routine intraoperative monitor in most hospitals, and it is one of the preferred instruments for bedside monitoring [7]. The Massimo ® pulse oximeter (Massimo Corp., Irvine, CA, USA) adds a module for monitoring of respiratory changes in the pulse oximetry plethysmographic waveform, derived from the perfusion index (PI) [8]. PI is defined as pulsatile and non-pulsatile tissues ratio of absorbed light. Pleth variability index (PVI) reflects the variation of PI in the respiratory cycle.

PVI can be continuously monitored on the display screen by connecting the probe of pulse oximeter. It is generated by the pulse oxygen probe and the absorption of red and infrared light at the measuring site.

Several trials have contributed to investigating the reliability of the PVI in predicting preload responsiveness [9–33]. On this basis, three system reviews evaluate the high accuracy of PVI [34–36]. A series of studies have shown that the PVI can reliably predict preload responsiveness during mechanical ventilation; however, some of these studies are not convincing because the sample size was less than 30 [11, 12, 17, 19, 29, 33]. Broch O et al. reported that the PVI reliably predicted preload responsiveness only in patients with high perfusion level (PI>4%) [9]. Le Guen et al. supported that the accuracy of PVI is limited during kidney transplantation [22]. Moreover, Maughan BC et al. indicated that PVI also cannot reliably predict preload responsiveness during cardiac surgery [26]. There seems to be no consensus on the reliability of PVI for different patients. The purpose of this review is to assess the reliability of the PVI to predict preload responsiveness in different mechanically ventilated patients (patients in different locations, with different types of surgery, different ages, and different methods of expansion).

## Methods and materials

### Search strategy

PUBMED, EMBASE, Cochrane Library, and Web of Science databases (last updated to November 7, 2018) were searched by two reviewers independently, using the keywords as follow: (plethysmography OR pleth OR plethysmographic) AND (variability OR variation) AND (index OR indices OR indexes). The references of all reviewed articles were viewed to look for valuable studies. Relevant authors and researchers had been contacted for complete data.

### Eligibility criteria

We included diagnostic trials that evaluated the reliability of the PVI to predict fluid responsiveness in patients with mechanical ventilation. We excluded reviews, case reports, comments, experiments on animals, or in vitro studies and articles that were not published in English.

### Quality assessment

Two reviewers independently assessed the quality of reviewed studies using the QUADAS-2 scale by Review Manager 5.3(Cochrane Library, Oxford, UK) [37]. Disagreement was resolved by discussion with third reviewer.

### Data extraction

The study characteristics and outcomes were examined and extracted by two reviewers independently. The following data were recorded using Microsoft Excel 2016 (Microsoft Corp, Redmond, WA): first author, year of publication, characteristics of patient, place of study, number of patients studied, tidal volume, amount of fluid infusion, the f value for defining responders to preload responsiveness, true positive rate, false positive rate, false negative rate, true negative rate, best cut-off value, sensitivity, specificity, the pooled area under the curve (AUC) of receiver operating characteristics (ROC) and r value.

For further data analysis, we also assessed the pooled sensitivity, pooled specificity, pooled AUC, Youden index (sensitivity plus specificity minus one) and 95% credibility interval (CI) of them.

### Statistical treatment

Data calculation and graphics synthesis was performed by Stata (version 14.0). Threshold effect and nonthreshold effect both will lead to heterogeneity. We used Spearman correlation coefficient (Mixed Model) to evaluate the threshold effect and used Cochrane-Q value of the AUC to evaluate nonthreshold effect. The heterogeneity was represented by the I^2^ statistic: when I^2^<25%, it means low heterogeneity exists, when 25%<I^2^<50%,it means moderate heterogeneity exists, and when I^2^ ≥ 50%, it means significant heterogeneity exists. Sensitivity analyses (test each article individually whether it is a source of heterogeneity) and meta-regression (patient’s surgeries; patient’s age; choice of patients volume expansion methods) were used to find the source of heterogeneity. We used Deeks’ Funnel Plot Asymmetry Test For Diagnostic Odds Ratio to determine whether significant publication bias exists in the articles included in the analysis [38].

## Results

### Literature search and study characteristics

**Fig. 1:**
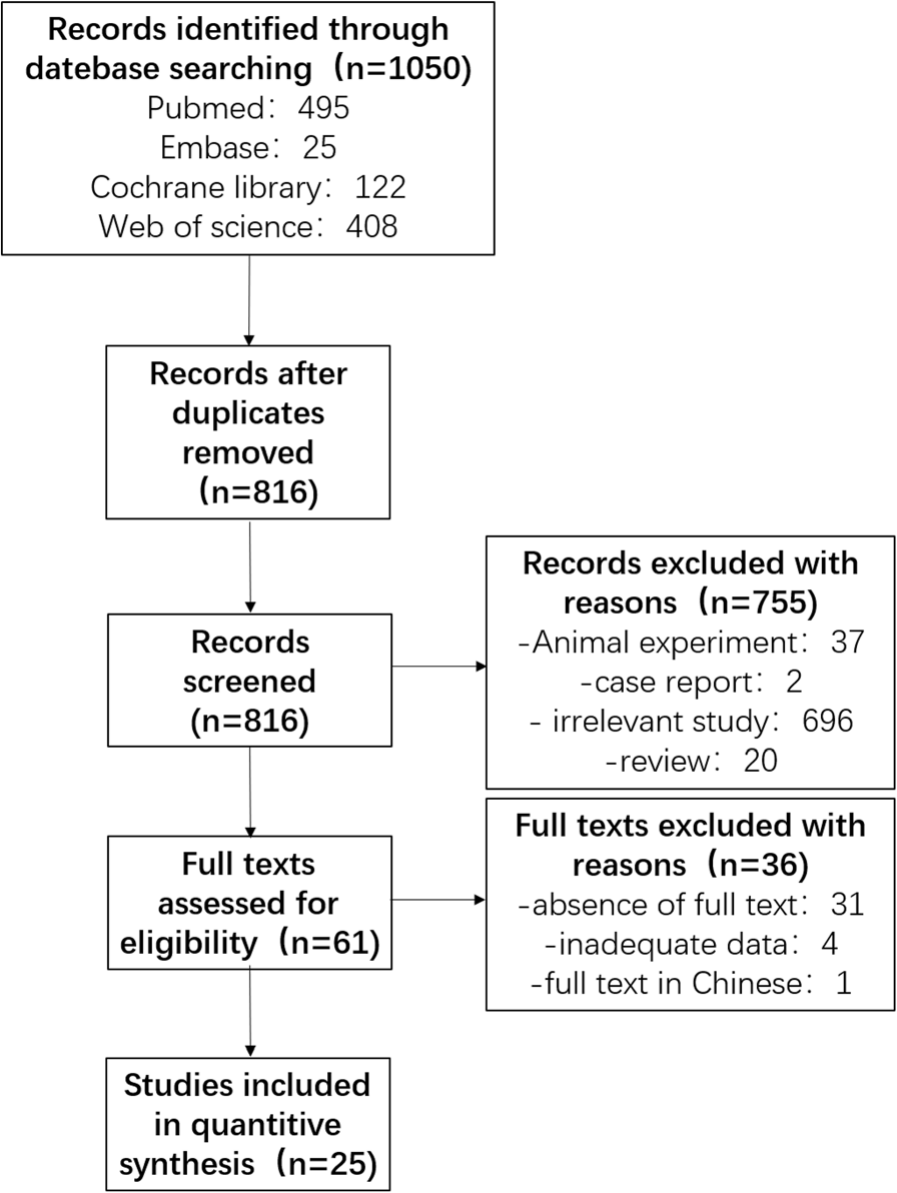
The search, inclusion and exclusion of the literature

The original literature search included 1068 articles, of which 1007 articles were excluded by reviewing title and abstracts because they were duplicates, irrelevant studies, animal experiments, conference summaries, case reports or review articles. After careful browsing of the remaining 61 studies, 31 studies were excluded because they lacked the full-text article. Four studies were excluded because the lack of relevant data on outcomes. One study was excluded because its abstract was published in English, while its full-text was published in Chinese. The retrieved, included and excluded articles for meta-analysis are summarized in Fig. 1. Characteristics of the 25 retrieved studies are summarized in Additional file 1.

### Quality assessment and publication bias

**Fig. 2:**
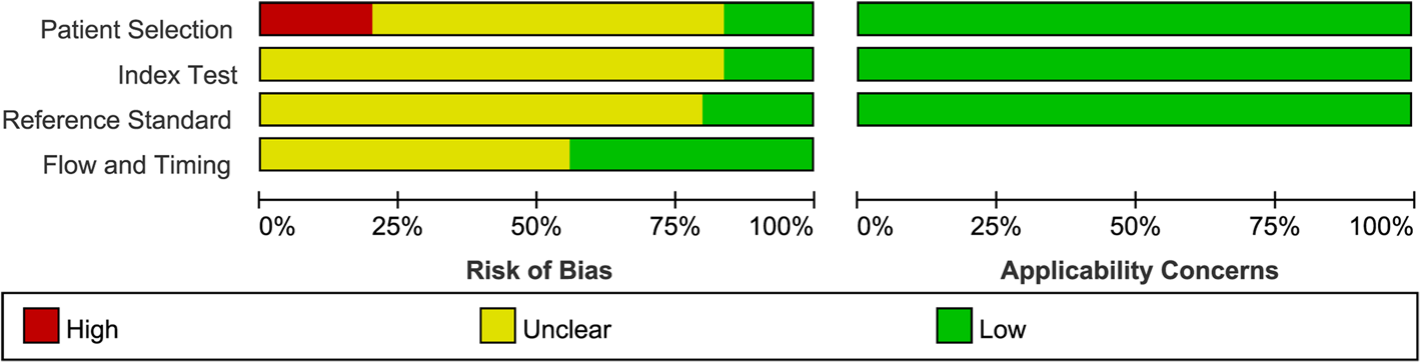
The results of quality assessment of the included articles (overview)

**Fig. 3:**
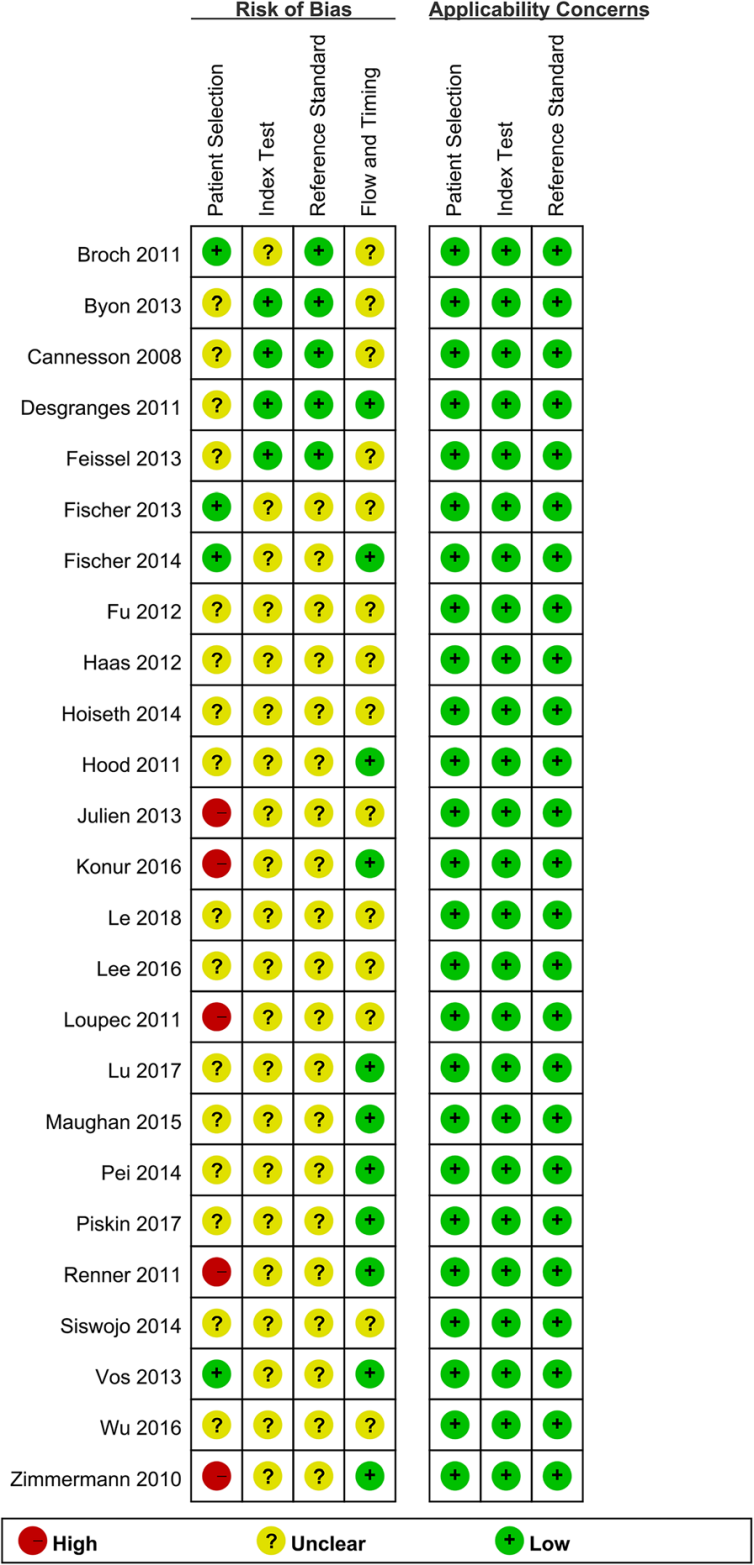
The results of quality assessment of each articles

Quality assessment of 25 retrieved studies is shown in Figs. 2 and 3.

The result of Deeks’ Funnel Plot Asymmetry Test for Diagnostic Odds Ratio is that the *P* value = 0.76, indicates that no significant publication bias exists in the included literature.

### Results of retrieved studies

The results of each retrieved studies are shown in Additional file 2. Twenty-five studies that included 1035 patients. The best cut-off value for PVI varied between 7 and 20%, while 1 study [18] did not provide information regarding the cut-off value. In 3 studies [20, 22, 32], the same patient receives more than one volume expansion, and the final data analysis uses the data for each volume expansion. Two studies [20, 23] evaluated preload responsiveness at two different period of surgery, so we divided the results of each study into two parts.

### Results of meta-analysis

**Fig. 4:**
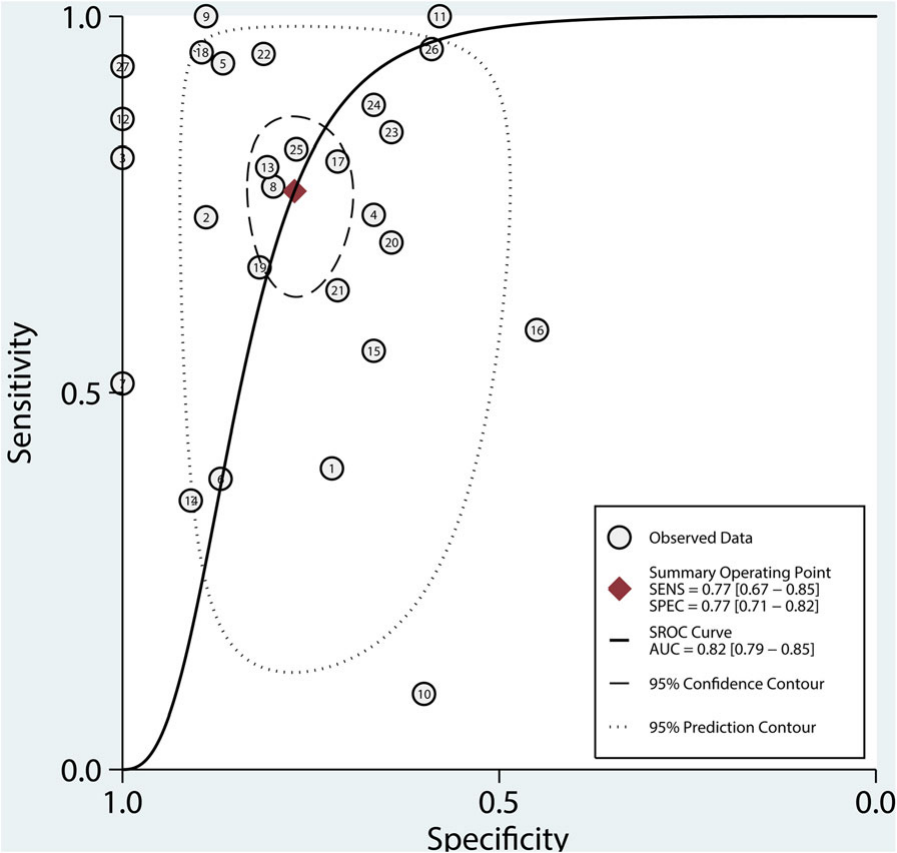
The summary receiver operating characteristics (SROC) of the included articles

The Spearman correlation coefficient was 0.07 (*P* < 0.01), indicates that although a significant threshold effect exists, the effect on the results is small. The Cochrane-Q value of the AUC was 39.175 (95% CI 0.79–0.85, *P* < 0.001) and I^2^ = 95%, indicates significant heterogeneity exists. Because of the significant heterogeneity of the pooled results, we performed a further subgroup analysis based on the patient’s condition. The results of the meta-analysis are described in Table 1 and Fig. 4. The pooled AUC was 0.82 (95% confidence interval (CI) 0.79–0.85). The pooled sensitivity was 0.77 (95% CI 0.67–0.85) and the pooled specificity was 0.77 (95% CI 0.71–0.82). The results shown that the accuracy of PVI predicting preload reactivity is not as high as reported in previous meta-analyses [34–36]. Our new discovery is the result of patients without undergoing surgery (AUC = 0.86, Youden index = 0.65) was reliable.

**Table 1:** Results of meta-analysis

Setting (numbers of studies)	Sensitivity(95% CI)	Specificity(95% CI)	Youden index	AUC(95% CI)-ROC	I^2^(%)
PVI across all settings(*n* = 27)	0.77 (0.67–0.85)	0.77 (0.71–0.82)	0.54	0.82 (0.79–0.85)	95
PVI in OR(*n* = 18)	0.76 (0.67–0.84)	0.76 (0.68–0.82)	0.52	0.82 (0.79–0.85)	81
PVI in ICU(*n* = 4)	0.79 (0.41–0.95)	0.88 (0.77–0.94)	0.67	0.89 (0.86–0.92)	89
PVI in adult(*n* = 22)	0.77 (0.65–0.85)	0.77 (0.70–0.82)	0.54	0.82 (0.79–0.85)	95
PVI in cardiac surgery(*n* = 9)	0.67 (0.40–0.87)	0.78 (0.66–0.87)	0.45	0.80 (0.77–0.84)	89
PVI in noncardiac surgery(*n* = 12)	0.78 (0.64–0.88)	0.71 (0.58–0.82)	0.49	0.80 (0.76–0.83)	63
PVI without surgery(*n* = 6)	0.85 (0.69–0.94)	0.80 (0.70–0.87)	0.65	0.86 (0.82–0.89)	33
PVI with colloid injection(*n* = 17)	0.77 (0.67–0.85)	0.82 (0.77–0.86)	0.59	0.83 (0.80–0.86)	87
PVI with crystalloid injection(*n* = 4)	0.77 (0.60–0.88)	0.69 (0.52–0.81)	0.46	0.79 (0.75–0.82)	23

Abbreviations: *AUC* area under the curve, *ROC* receiving operating characteristics

### Heterogeneity

The pooled I^2^ value was 95%, indicating statistically significant heterogeneity. After performance of meta-regression, we found the choice of intravenous colloid injection as a means of preload responsiveness was a significant cause (*p* = 0.02) of the heterogeneity; however, following the exclusion of the 17 studies which used intravenous colloid injection [10–16, 19–21, 23, 24, 29–33], the heterogeneity remained significant(I^2^ = 84%).

The sensitivity analysis showed that 2 [16, 27] of the studies may have contributed to the heterogeneity; however, following the exclusion of the two studies, the heterogeneity remained significant(I^2^ = 95%).

Significant heterogeneity exists in both the overall group and most of the subgroups, which may be because of patient’s complex conditions, different surgical methods and the different fluid management methods. The heterogeneity was relatively low in the subgroup of patients undergoing noncardiac surgery(I^2^ = 63%), which may be because of the patients undergoing cardiac surgery are often non-sinus rhythms and have a greater impact on tissue perfusion. No significant heterogeneity exists in the subgroups of patients without undergoing surgery (I^2^ = 33%), which may be because certain surgical stimuli (such as pain) and procedures (such as liver surgery for inferior vena cava) may cause changes in vascular tension or hemodynamics. No significant heterogeneity exists in the crystalloid subgroup (I^2^ = 23%), potentially because of the small number of studies (*n* = 4).

With the data emerging from our meta-analysis, no certain assertion can be made. The study provides interesting data and the results of the subgroups of patients without undergoing surgery should be reliable.

## Discussion

### Applicable patients

The PVI has higher accuracy for mechanically ventilated patients with a regular rhythm and nonthoracotomy [39]. The PVI reflects the degree of change in PI caused by breathing over a period of time, so PVI is greatly affected by cardiopulmonary exercise. The PVI has ability to reliably predict preload responsiveness, provided that the pressure changes in the chest cavity are sufficiently obvious enough and the cardiopulmonary interaction between different respiratory cycles is stable. Therefore, the PVI and other dynamic parameters of cardiopulmonary interaction are more suitable for patients with mechanical ventilation rather than spontaneous breathing. The results of the meta-analysis also showed that PVI was less reliable in the subgroup of cardiac surgery (Youden index =0.45) than in the non-cardiac surgery subgroup (Youden index =0.49).

### Perfusion situation

Under the monitoring of a pulse oximeter, the pulsating blood flow absorbs red and infrared light (AC), and the tissue and skin also absorb red and infrared light (DC). The ratio of the two parameter can calculate the PI:

$$ \mathrm{PI}=\left(\mathrm{AC}-\mathrm{DC}\right)\times 100\% $$

PVI reflects the degree of change in PI caused by breathing over a period of time. The formula is as follows:

$$ \mathrm{PVI}=\left[\left({\mathrm{PI}}_{\mathrm{max}}-{\mathrm{PI}}_{\mathrm{min}}\right)/{\mathrm{PI}}_{\mathrm{max}}\right]\times 100\% $$

Reliability of the PVI is largely affected by adequacy of perfusion [40]. Peripheral perfusion deficiency can result in impaired blood flow to a stable constant partly caused by skin and other factors that signal the volume in the tissue. To date, a pulsed oximeter, which is used to calculate the PVI, will not be able to determine whether the reduction of chest pressure is caused by the variety of cardiovascular system capacity or low perfusion of the monitored site, so any influence on peripheral perfusion factors, that is, the factors that affect PI, can affect the reliability of the PVI [34]. The sensitivity of the subgroup of cardiac surgery is lower than that of the other subgroups and overall (0.67 95% CI 0.40–0.87). Broch O et al. [9] reported that the PVI reliably predicted preload responsiveness only in patients with high perfusion level (PI>4%).

When using the PVI to guide goal-directed volume expansion, anesthetists should pay attention to factors that can affect perfusion situation of the monitored site (such as peripheral vascular disease, severe heart failure, application of vasoactive drugs, and damage of the monitored site).

### Types of volume expansion

The results of the synthesis show that the subgroups with colloid injection (Youden index = 0.59 AUC = 0.83) are more reliable than the subgroups with crystalloid injection (Youden index = 0.46 AUC = 0.79). This may be because the colloidal fluid has a better effect on the macrocirculation and the microcirculation [41], thus increasing the reliability of the PVI.

### The best cut-off value

The included results show that the PVI has a wide range of best cut-off value for defining responders to preload responsiveness, which range from 7 to 20%. The different conditions for each study (the patients’ underlying disease, volume stroke, age, type of surgery, in operating room or in ICU), and patients’ different fluid management (the application of vasoactive drugs, rate of intravenous infusion and type of volume expansion) may contribute to high variability. We suggest that readers can refer to the cut-off values reported in the corresponding articles when applying PVI to different patients.

### Monitored site

The monitored site can affect the morphology and respiratory variation of the PVI [42–45]. Desgranges et al. [12] compared finger, forehead and ear as monitored site, reporting that the choice of three monitored sites has no significant impact on accuracy. While Hood et al. [19] reported that the PVI_finger_ can reliably predict increases in SV, while the PVI_earlobe_ can not reliably predict increases in SV in dynamic intraoperative conditions. Fischer et al. [15] demonstrated PVI_forehead_ was more accurate than PVI_finger_ in patients after cardiac surgery. For safety and convenience, the PVI_finger_ remains the preferred choice for most patients, with the PVI_forehead_ and PVI_earlobe_ as stable alternatives [12].

### Limitations

Our systematic review has several limitations. First, significant heterogeneity exists in both the overall group and most subgroups; thus, differences between patients and surgeries should be considered in the application of the PVI. Second, we only included mechanically ventilated patients, which limited the results extrapolated to all patients. Studies on the monitoring of the PVI on patients with spontaneous breathing must be conducted. Third, subgroup analyses of the child subgroup and the passive leg raise subgroup were not performed because of insufficient studies. Fourth, the best cut-off value for the PVI varied within great ranges, and the best cut-off value for different types of patients and surgeries remains to be studied. Finally, although PVI is more reliable for patients in the ICU, most of these patients are also applying other more accurate invasive monitoring (such as arterial blood pressure monitoring), so PVI is more recommended as a supplement of pulse oxygen.

## Conclusion

The PVI, as a noninvasive and automatic hemodynamic monitoring, has limited ability to predict the fluid responsiveness of mechanically ventilated patients, except patients without undergoing surgery and patients in ICU. The PVI can plays an important role in bedside monitoring for mechanically ventilated patients who are not undergoing surgery, such as patients after cardiac surgery and shock patients. Patients who are expanded with colloid may be more suitable for PVI. For different individuals, the optimal PVI cut-off value must be further determined.

## Additional files

Additional file 1: Characteristics of the retrieved studies, including published year, setting, type and mean age of patients, sample size, type of fluid challenge and definition of responsiveness of included studies. (DOCX 50 kb)

Additional file 2: Results of the retrieved studies, including sample size, true positive, false positive, false negative, true negative, best cut-off value, sensitivity, specificity, AUC and r value of included studies. (DOCX 20 kb)

## Abbreviations

AUC: Area under the curve
CI: Confidence interval
ICU: Intensive care unit
PI: Perfusion index
PVI: Pleth Variability Index
