# Supplementary material for: Reliability of pleth variability index in predicting preload responsiveness of mechanically ventilated patients under various conditions: a systematic review and meta-analysis
Source: BMC Anesthesiol. 2019 May 8;19:67. doi: 10.1186/s12871-019-0744-4 (PMC6507157; doi:10.1186/s12871-019-0744-4)
Supplement: Supplementary file 1 — Characteristics of the retrieved studies, including published year, setting, type and mean age of patients, sample size, type of fluid challenge and definition of responsiveness of included studies. (DOCX 50 kb) [file 12871_2019_744_MOESM1_ESM.docx]

**Additional file 1 Characteristics of the retrieved studies**

| Study | Year | Setting | Type of patient | Size | Mean age（year） | Fluid challenge | Definition of responsiveness |
| --- | --- | --- | --- | --- | --- | --- | --- |
| Broch[11] | 2011 | OR | adult before elective coronary artery surgery | 81 | 66 | passive leg raise | ΔSVII≥15% |
| Byon[12] | 2013 | OR | children-neurosurgery | 37 | 6 | colloids 10 ml/kg | ΔSVI≥10% |
| Cannesson[13] | 2008 | OR | adult-coronary artery bypass grafting | 25 | 65 | colloids 500 ml | ΔCI≥15% |
| Desgranges[14] | 2011 | OR | adult-cardiac surgery | 28 | 62 | colloids 500 ml | ΔCI≥15% |
| Feissel[15] | 2013 | ED | adult with septic shock | 39 | 65 | colloids 8 ml/kg | Δaortic velocity–time integral ≥15% |
| Fischer[16] | 2013 | ICU | adult-postoperation, cardiac surgery | 87 | 71 | colloids 500 ml | ΔCI≥15% |
| Fischer[17] | 2014 | ICU | adult-postoperation, cardiac surgery | 50 | 73 | colloids 500 ml | ΔCI≥15% |
| Fu[18] | 2012 | OR | adult-resection of primary retroperitoneal tumors | 55 | 48.7 | colloids 8 ml/kg | ΔSVI≥10% |
| Haas[19] | 2012 | OR | adult-cardiac surgery after cardiopulmonary bypass | 18 | 67.5 | colloids 4 ml/kg | ΔCI≥10% |
| Hoiseth[20] | 2014 | OR and ICU | adult before undergoing aortic valve replacement | 32 | 70 | 4 for crystalloids 500 ml 12 for colloids 250 ml 12 for PRBC 250 ml 3 for Plasma 250 ml | ΔSV≥15% |
| Hood[21] | 2011 | OR | adult-colorectal resection | 25 | 62 | colloids 500 ml | ΔSV≥10% |
| Julien[22] | 2013 | OR | children-noncardiac surgery | 54 | 4 | crystalloids 10 ml/kg | ΔSVI≥15% |
| Konur[23] | 2016 | OR | adult-orthotopic liver transplantation | 25 | 49.32 | colloids 10 ml/kg | ΔCI≥15% |
| Le[24] | 2018 | OR | adult-kidney transplantation | 48 | 54 | crystalloids 250 mg | ΔSV≥10% |
| Lee[25] | 2016 | OR | adult-arthroscopic shoulder procedures | 42 | 62 | colloids 6 ml/kg | ΔSVI≥15% |
| Loupec[26] | 2011 | ICU | adult with circulatory insufficiency | 45 | 58 | colloids 500 ml | ΔCO≥15% |
| Lu[27] | 2017 | - | adult with septic shock | 49 | 55.9 | crystalloids 200 ml | ΔCI≥10% |
| Maughan[28] | 2015 | - | adult-postoperation, cardiac surgery | 47 | 70 | passive leg raise | ΔCI≥15% |
| Pei[29] | 2014 | OR | adults with obstructive jaundice | 32 | 54.8 | colloids 250 ml | ΔSVI≥10% |
| Piskin[30] | 2017 | ICU | adult | 72 | 64.38 | passive leg raise | ΔCI≥15% |
| Renner[31] | 2011 | OR | infants-congenital heart surgery | 27 | 1.42 | colloids 10 ml/kg | ΔSVI≥15% |
| Siswojo[32] | 2014 | OR | adults-noncardiac surgery | 30 | 55 | colloids 500 ml | ΔSVI≥10% |
| Vos[33] | 2013 | OR | patients-major hepatic resection | 30 | 56 | colloids 15 ml/kg (n=15) or crystalloids 15 ml/kg (n=15) | ΔSVI≥20% |
| Wu[34] | 2016 | OR | adult-living donor orthotopic liver transplant | 37 | 53 | crystalloids 10 ml/kg | ΔSV≥15% |
| Zimmermann[35] | 2010 | OR | adults-major abdominal surgery | 20 | 53 | colloids 7 ml/kg | ΔSVI≥15% |

Abbreviations: OR，operating room; ICU, intensive care unit; ED, emergency department; CI, cardiac index; CO, cardiac output; SVI, stroke volume index; SV, stroke volume; colloids, intravenous colloid injection; crystalloids, intravenous crystalloids injection.
