# Supplementary material for: Reliability of pleth variability index in predicting preload responsiveness of mechanically ventilated patients under various conditions: a systematic review and meta-analysis
Source: BMC Anesthesiol. 2019 May 8;19:67. doi: 10.1186/s12871-019-0744-4 (PMC6507157; doi:10.1186/s12871-019-0744-4)
Supplement: Supplementary file 2 — Results of the retrieved studies, including sample size, true positive, false positive, false negative, true negative, best cut-off value, sensitivity, specificity, AUC and r value of included studies. (DOCX 20 kb) [file 12871_2019_744_MOESM2_ESM.docx]

**Additional file 2 Results of the retrieved studies**

| Study | Size(n） | TP | FP | FN | TN | Best cut-off value (%) | Sensitivity | Specificity | AUC（CI95%）[p]-ROC | r |
| --- | --- | --- | --- | --- | --- | --- | --- | --- | --- | --- |
| Broch [11] | 81 | 18 | 10 | 27 | 26 | 14% | 41% | 72% | 0.60(0.47-0.72) [0.11] | 0.29 |
| Byon [12] | 33*^b^* | 11 | 2 | 4 | 16 | 11% | 73.30% | 86.70% | 0.767(0.597–0.936) | 0.49 |
| Cannesson [13] | 25 | 13 | 0 | 3 | 9 | 14% | 81% | 100% | 0.927 (0.828-1.026) [<0.01] | 0.67 |
| Desgranges [14] | 28 | 14 | 3 | 5 | 6 | 12% | 74% | 67% | 0.836 (0.685-0.988) [0.005] | NA |
| Feissel [15] | 31*^c^* | 15 | 2 | 1 | 13 | 19% | 94% | 87% | 0.97（0.83-0.99）[<0.001] | NA |
| Fischer 2013 [16] | 80*^d^* | 22 | 3 | 35 | 20 | 20% | 38% | 87% | 0.60 (0.48-0.71) [ 0.020] | -0.22 |
| Fischer 2014 [17] | 50 | 21 | 0 | 20 | 9 | 19% | 51% | 100% | 0.74（0.60-0.86) | NA |
| Fu [18] | 51*^e^* | 24 | 4 | 7 | 16 | 13.50% | 77.40% | 80% | 0.785 (0.651-0.920) [0.002] | 0．362 |
| Haas [19] | 22*^a^* | 4 | 2 | 0 | 16 | 16% | 100% | 88.90% | 0.95[0.31] | 0.80 |
| Hoiseth (before replacement） [20] | 30*^f^* | 2 | 4 | 18 | 6 | 53% | 11% | 60% | 0.51 (0.31–0.69) | NA |
| Hoiseth (after replacement） [20] | 31*^g^* | 12 | 8 | 0 | 11 | 80% | 100% | 59% | 0.72 (0.52–0.87) | NA |
| Hood [21] | 25 | 19 | 0 | 3 | 3 | 10% | 86% | 100% | 0.96 (0.88–1.00) [0.011] | NA |
| Julien [22] | 97*^a^* | 36 | 10 | 9 | 42 | 17.00% | 80% | 80% | 0.85（0.77-0.93） | NA |
| Konur(Dissection phase）[23] | 25 | 5 | 1 | 9 | 10 | 7% | 35% | 90% | 0.56[0.58] | 0.34 |
| Konur(Anhepatic phase）[23] | 25 | 10 | 2 | 8 | 4 | 16% | 55% | 66% | 0.55[0.58] | NA |
| Le [24] | 44*^h^* | 14 | 11 | 10 | 9 | NA | 59% | 47% | 0.49(0.36-0.62) [0.83] | NA |
| Lee [25] | 40*^i^* | 21 | 4 | 5 | 10 | 10% | 80% | 70% | 0.79（0.56–0.92) [0.011] | NA |
| Loupec [26] | 40*^j^* | 20 | 2 | 1 | 17 | 17% | 95.00% | 91% | 0.88（0.74-0.96） | 0.72 |
| Lu [27] | 49 | 18 | 4 | 9 | 18 | 15.50% | 65% | 80% | 0.816 (0.686 - 0.946) [0.001] | NA |
| Maughan [28] | 24*^k^* | 7 | 5 | 3 | 9 | 14% | 67% | 67% | 0.63[0.16] | NA |
| Pei [29] | 32 | 7 | 6 | 4 | 15 | 15% | 63.60% | 71.40% | 0.567 (0.381-0.740) [0.54] | NA |
| Piskin [30] | 72 | 38 | 6 | 2 | 26 | 14% | 95% | 81.20% | 0.939（0.857-0.982）[<0.001] | NA |
| Renner [31] | 27 | 11 | 5 | 2 | 9 | 13% | 84% | 61% | 0.79(0.61–0.88) [0.01] | NA |
| Siswojo [32] | 29*^l^* | 15 | 4 | 2 | 8 | 10.50% | 88% | 67% | 0.84 (0.69-0.99) | NA |
| Vos [33] | 30 | 14 | 3 | 3 | 10 | 12% | 82% | 77% | 0.78 (0.59–0.96) | NA |
| Wu [35] | 62 | 22 | 16 | 1 | 23 | 11% | 95.70% | 59% | 0.8（0.679-0.891） | -0.44 |

Abbreviations: AUC, area under the curve; FN, false negatives; FP, false positives; NA, not available; ROC, receiving operating characteristics; TN, true negatives; TP, true positives.
